# Enhanced methane production from cellulose using a two-stage process involving a bioelectrochemical system and a fixed film reactor

**DOI:** 10.1186/s13068-020-01866-x

**Published:** 2021-01-06

**Authors:** Kengo Sasaki, Daisuke Sasaki, Yota Tsuge, Masahiko Morita, Akihiko Kondo

**Affiliations:** 1 Graduate School of Science, Technology and Innovation, Kobe University, 1-1 Rokkodaicho, Nada-ku, Kobe, Hyogo 657-8501 Japan; 2 Institute for Frontier Science Initiative, Kanazawa University, Kakuma-machi, Kanazawa, Ishikawa 920-1192 Japan; 3 Environment Chemistry Sector, Environmental Science Research Laboratory, Central Research Institute of Electric Power Industry, 1646 Abiko, Abiko-shi, Chiba-ken 270-1194 Japan; 4 RIKEN Center for Sustainable Resource Science, 1-7-22 Suehiro-cho, Tsurumi-ku, Yokohama, Kanagawa 230-0045 Japan

**Keywords:** Bioelectrochemical system, Fixed film reactor, Two-stage process, Methane, Hydrogenotrophic methanogen, Cellulose

## Abstract

**Background:**

It is desirable to improve the anaerobic digestion processes of recalcitrant materials, such as cellulose. Enhancement of methane (CH_4_) production from organic molecules was previously accomplished through coupling a bioelectrochemical system (BES); however, scaling-up BES-based production is difficult. Here, we developed a two-stage process consisting of a BES using low-cost and low-reactive carbon sheets as the cathode and anode, and a fixed film reactor (FFR) containing conductive material, i.e., carbon fiber textiles (CFTs) (:BES → FFR). By controlling the cathodic current at 2.7 μA/cm^2^ without abiotic H_2_ production, the three-electrode BES system was operated to mimic a microbial electrolysis cell.

**Results:**

The thermophilic BES (inlet pH: 6.1) and FFR (inlet pH: 7.5) were operated using hydraulic retention times (HRTs) of 2.5 and 4.2 days, respectively, corresponding to a cellulose load of 3555.6 mg-carbon (C)/(L day). The BES → FFR process achieved a higher CH_4_ yield (37.5%) with 52.8 vol% CH_4_ in the product gas compared to the non-bioelectrochemical system (NBES) → FFR process, which showed a CH_4_ yield of 22.1% with 46.8 vol% CH_4_. The CH_4_ production rate (67.5 mM/day) obtained with the BER → FFR process was much higher than that obtained using electrochemical methanogenesis (0.27 mM/day). Application of the electrochemical system or CFTs improved the yields of CH_4_ with the NBES → FFR or BES → non-fixed film reactor process, respectively. Meta 16S rRNA sequencing revealed that putative cellulolytic bacteria (identified as *Clostridium* species) were present in the BES and NBES, and followed (BES→ and NBES→) FFR. Notably, H_2_-consuming methanogens, *Methanobacterium* sp. and *Methanosarcina* sp., showed increased relative abundances in the suspended fraction and attached fraction of (BES→) FFR, respectively, compared to that of (NBES→) FFR, although these methanogens were observed at trace levels in the BES and NBES.

**Conclusions:**

These results indicate that bioelectrochemical preprocessing at a low current effectively induces interspecies H_2_ transfer in the FFR with conductive material. Sufficient electrochemical preprocessing was observed using a relatively short HRT. This type of two-stage process, BES → FFR, is useful for stabilization and improvement of the biogas (CH_4_) production from cellulosic material, and our results imply that the two-stage system developed here may be useful with other recalcitrant materials.

## Background

Anaerobic digestion is one of the most promising biotechnologies for decomposition and stabilization of diverse organic substrates [1]. Currently, anaerobic digestion processes are frequently employed to produce renewable bioenergy in the form of biogas, such as CH_4_, which can be used as a replacement for fossil fuels to generate heat or electricity [2]. An important challenge of using anaerobic digesters is enhancing the overall process stability and achieving consistent biogas production, with a high percentage of substrate utilization. Many efforts have been made to control the various microorganisms involved in the multi-stage biochemical processes, including hydrolysis, acidogenesis, acetogenesis, and methanogenesis [3]. To improve the efficiency of digestion by microbial retention, various reactor types have been developed, such as fixed film reactors (FFRs) (previously referred to as packed-bed reactor in our study) and fluidized bed reactors [4]. Additionally, two-stage processes to separate the acidogenic phase and methanogenic phases have been proposed [5] to increase total biogas production and demonstrate better process stability than single-stage processes [6].

An bioelectrochemical system (BES) uses microorganisms that transfer electrons to an electrode or that receive electrons from the electrode, which can be used for applications, such as wastewater treatment with simultaneous electricity production, electrochemical production of hydrogen (H_2_) or methane (CH_4_), and desalination [7]. A combination of anaerobic digestion and BES was previously used to develop a more robust waste treatment design [8]. Essentially, an electrochemical system consists of an anode and cathode, optionally separated by an ion-selective membrane to create two separate compartments through which a current can be applied or produced spontaneously; however, using a membrane may add to the overall cost of the system [9]. Thus, in many cases, bioelectrochemically assisted anaerobic digestion is performed without a membrane, and the externally applied potential can enhance CH_4_ production, which makes in situ biogas upgradation possible [10–12]. This may be due to alterations in microbial metabolism and interspecies interactions, particularly affecting hydrogenotrophic methanogens [13, 14]; because the H_2_ concentration, i.e., H_2_ consumption by methanogens, influences the biodegradation of organic compounds [15]. The electrode surface area and material are limiting factors for scaling up an integrated anaerobic digester and a BES [8]; however, anaerobic digesters are configured for large-scale production. Therefore, in this study, the BES and the anaerobic digester were separated and operated in a two-stage process. Moreover, we aimed to extend the effects of the first stage (the BES) to the second stage (an anaerobic digester using a fixed film).

In this study, a three-electrode BES was used in the first stage as a microbial electrolysis cell mode, where abiotic H_2_ production was inhibited at a low current of 2.7 μA/cm^2^ with low-cost and low-reactive carbon (C) electrodes, for both the cathode and anode. The main purpose of this design strategy was to boost CH_4_ production, particularly in the latter FFR stage, which involved fixation of conductive carbon fiber textiles (CFTs) using a two-stage process (BES → FFR). Thus, inlet pH (pH_in_) in the latter FFR or non-fixed film reactor (NFFR) stage was set to a neutral pH (7.5); however, the pH_in_ in the BES or non-bioelectrochemical system (NBES) was set to an acidic pH (pH 6.1) to suppress biogas production in the former stage (Table 1). The NBES (without an external electrochemical system) and NFFR (without CFTs) were used as control reactors for BES and FFR, respectively. We propose utilization of a BES–FFR combination reactor to efficiently recover biogas from a model substrate containing cellulose as the major C source. Cellulose biodegradation has been evaluated because cellulose is the rate-limiting substrate in the anaerobic digestion [16].

**Table 1:** Operating conditions used for different two-stage processes

Reactor type	HRT (days)	pH_in_	Aim
BES → FFR	4.0 → 8.3	6.1 → 7.5	Acclimation for 17 days
BES → FFR	2.5 → 4.2	6.1 → 7.5	Comparison after 13 days
NBES → FFR	4.0 → 8.3	6.1 → 7.5	Acclimation for 17 days
NBES → FFR	2.5 → 4.2	6.1 → 7.5	Comparison after 13 days
BES → NFFR	4.0 → 8.3	6.1 → 7.5	Acclimation for 17 days
BES → NFFR	2.5 → 4.2	6.1 → 7.5	Comparison after 13 days

NBES did not contain an electrochemical system. The FFR contained CFTs, whereas the NFFR did not contain CFTs

## Results

### Bioelectrochemical preprocessing accelerates methanogenesis from cellulose

**Fig. 1:**
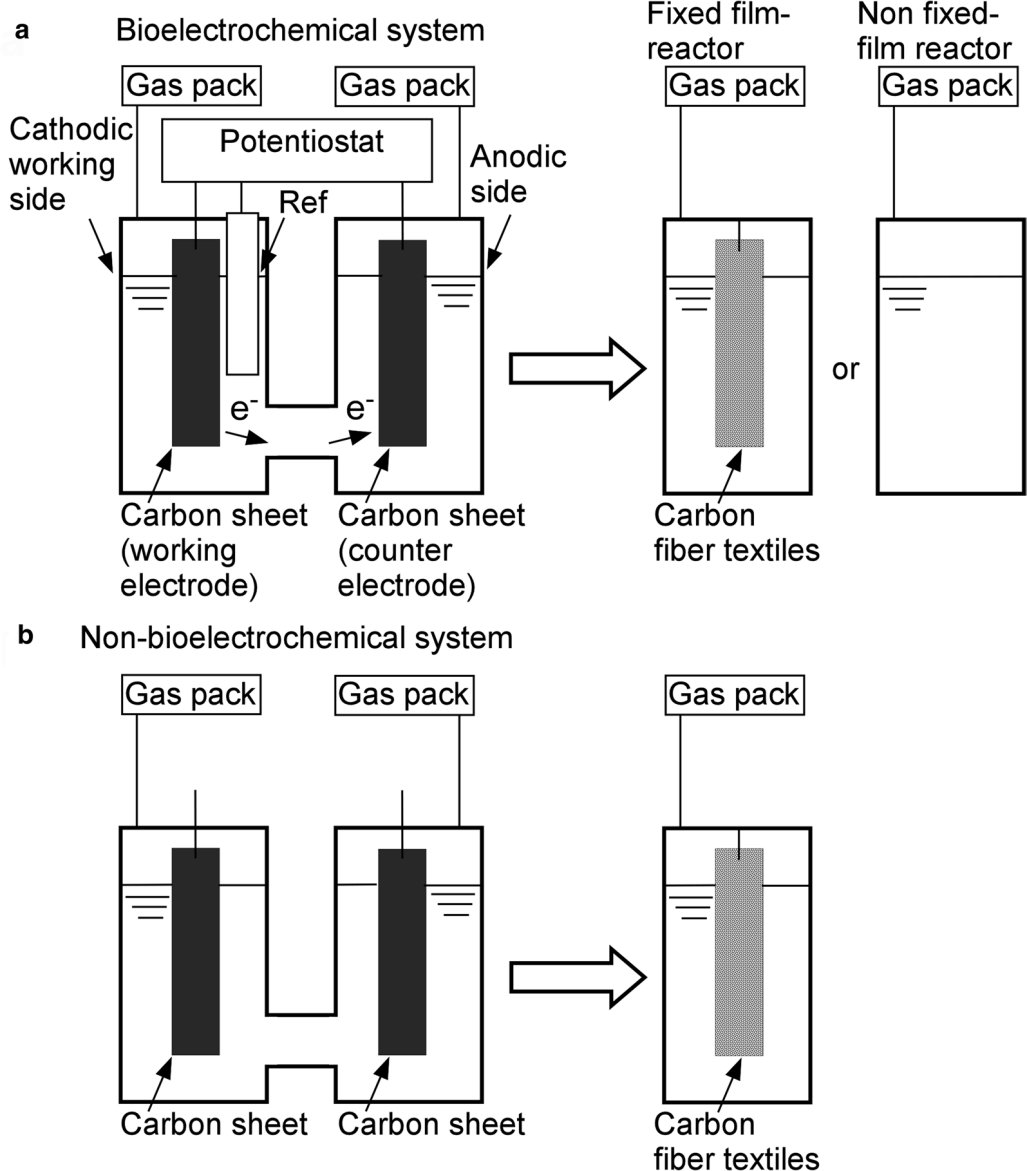
Schematic representation of the two-stage processes used in this study. The two-stage processes included **a** the BES followed by an FFR or an NFFR without CFTs, or **b** an NBES without a potentiostat followed by an FFR

First, we compared microbial CH_4_ production from the BES followed by the FFR (BES → FFR) with that from the NBES followed by FFR (NBES → FFR) (Fig. 1). In this study, the less-reactive C sheet (i.e., a graphite block) was used as the electrode, and the current of the cathodic working electrode in the BES was set to 2.7 μA/cm^2^, low enough to eliminate abiotic H_2_ production. This design enabled us to investigate the impact of electrochemical reactions on microbial CH_4_ production from cellulose substrates in the two-stage process. Both types of two-stage processes (BES → FFR and NBES → FFR) were performed for 17 days [twice the hydraulic retention time (HRT) of FFR] to acclimate the system to relatively long HRTs and establish a microbial consortium on CFT. Biofilms were rarely formed on the electrodes in BES and NBES. The results were compared with those found after 13 days of operation at a relatively short HRT (Table 1).

**Fig. 2:**
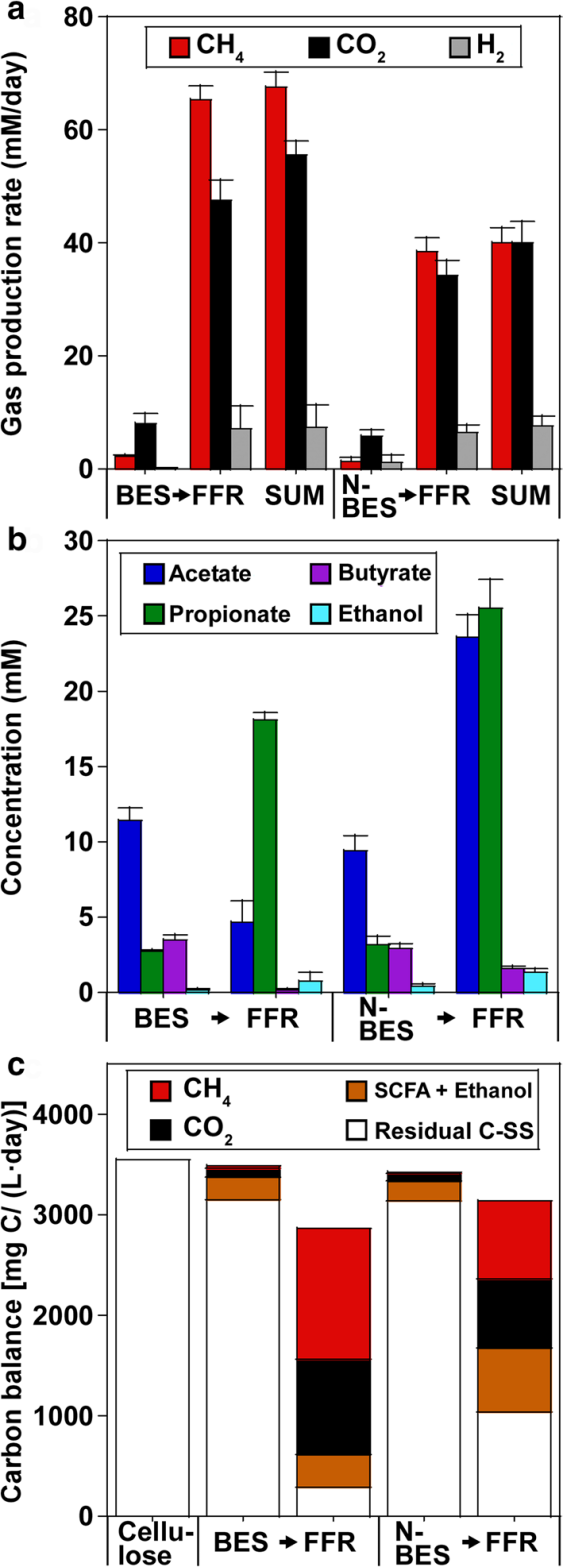
Performances of the two-stage BES → FFR and NBES → FFR processes using HRTs of 2.5 → 4.2 days. **a** Rates of gas production in terms of CH_4_, CO_2_, and H_2_. **b** Production of SCFAs and ethanol. **c** C balances of the two-stage processes. The cellulose loads of the BES and NBES were both 3555.6 mg C/(L day). The residual C in all reactors was calculated based on the SSs

The reactor performances were compared after the former and latter stages were operated at an HRT of 2.5 and 4.2 days, respectively (Fig. 2). The reactor performances were similar at both sides of the H-type reactors in the BES (the cathodic and anodic sides) and NBES (the left and right sides); therefore, the average values of both sides are reported for the BES and NBES in the present study. At this HRT (2.5 days), the synthetic medium containing cellulose as the major C source was added to the BES and NBES, at a cellulose load of 3555.6 mg C/(L day) (Fig. 2c). During the operation, pH_in_ (pH 6.1) decreased, and the outlet pH was 5.2 ± 0.3 and 5.2 ± 0.2 in the BES and NBES, respectively. Therefore, due to the low pH, most cellulose remained without being consumed, corresponding to the removal of 11.4 ± 4.3% and 11.6 ± 4.3% of the suspended solids (SSs) in BES and NBES, respectively. Biogas production was suppressed (Fig. 2a), and most of the microbial products were represented by short-chain fatty acids (SCFAs), such as acetate, propionate, and butyrate, and ethanol (Fig. 2b).

The effluents of the BES and NBES were added to the FFR, which implied that the residual C (consisting of SSs and SCFAs + ethanol) was the influent for the FFR (Fig. 2c). During the operation, the pH_in_ (pH 7.5) dropped, and the outlet FFR pH values were 6.8 ± 0.0 and 6.4 ± 0.4 with the [(BES→) FFR] and [(NBES→) FFR] systems, respectively. Interestingly, SS removal in the (BES→) FFR system (90.7 ± 2.2%) was much higher than that in the (NBES→) FFR system (66.9 ± 1.1%). Accordingly, the rate of biogas production (including CH_4_) was much higher in the (BES→) FFR system than in the (NBES→) FFR system (Fig. 2a). The SCFAs and ethanol concentrations were lower in (BES→) FFR than that in (NBES→) FFR. The efficient removal of intermediates from the fermentation process enhanced SS removal in (BES→) FFR. As a result, the two-stage BES → FFR process achieved a CH_4_ yield of 37.5 ± 1.4% with 52.8 ± 0.3 vol% CH_4_ in the product gas, whereas the NBES → FFR process demonstrated a 22.1 ± 1.4% CH_4_ yield with a 46.8 ± 1.3 vol% CH_4_ in the product gas.

### Microbial 16S rRNA gene sequence analysis of the microbial consortium in the reactors

The microbial communities developed during the two-stage processes, BES → FFR and NBES → FFR, were examined at an HRT of 2.5 and 4.2 days, respectively (Table 1). Microbial abundances were determined by real-time polymerase chain reaction (PCR) analysis (Table 2). The microbial abundances were low during the first stages (BES and NBES) due to low-pH conditions involved. The microbial abundances increased in the second stages, i.e., (BES→) FFR and (NBES→) FFR; however, the suspended microbial abundances in the (BES→) FFR process were relatively higher than those in the (NBES→) FFR process. With both the (BES→) FFR and (NBES→) FFR processes, the microorganisms were highly concentrated in fractions attached to the CFTs [(BES→) FFR-C and (NBES→) FFR-C].

**Table 2:** 16S rRNA gene copy numbers in the suspended fraction of reactors [BES-cathode, BES-anode, (BES→) FFR, NBES-left, NBES-right, and (NBES→) FFR] and CFT-attached fraction [(BES→) FFR-C and (NBES→) FFR-C]

	Eubacteria (copies/mL or copies/cm^2^-CFT)
BES-cathode	0.966 × 10^10^
BES-anode	0.998 × 10^10^
(BES →) FFR	13.0 × 10^10^
(BES →) FFR-C	12.8 × 10^10^
NBES-left	0.528 × 10^10^
NBES-right	0.649 × 10^10^
(NBES →) FFR	4.92 × 10^10^
(NBES →) FFR-C	14.0 × 10^10^

Using a combination of prokaryotic universal primers and the MiSeq platform, we obtained an average of 196,444 (± 29,391) reads for each sequencing reaction (Table 3). The number of operational taxonomic units (OTUs) describes the approximate number of species. OTU numbers in the second stages, (BES→ and NBES→) FFRs, were higher than those in the first stages, BES and NBES, due to pH differences. Microbial species diversity is represented by the Shannon index [17], and Faith’s phylogenetic diversity correlates with species richness [18]. The Shannon index value of the (BES→) FFR process was higher than that of the BES; however, the Shannon index value was lower with (NBES→) FFR than that with NBES. Consequently, the Shannon index value of (BES→) FFR process was higher than that of the (NBES→) FFR process for both suspended and attached fractions. Faith’s phylogenetic diversity values in the second stages, (BES→ and NBES→) FFRs, were higher than the values in the first stages, BES and NBES. For (BES→) FFR, the α-diversity values, Shannon index, and Faith’s phylogenetic diversity values in the fraction attached to CFT [(BES→) FFR-C] were higher than the values in the suspended fraction [(BES→) FFR].

**Table 3:** Summary of the 16S rRNA gene sequencing data and α-diversity values (Shannon index and Faith’s phylogenetic diversity)

	Read counts	Observed OTUs	Shannon index	Faith’s phylogenetic diversity
BES-cathode	201,720	55	2.67	5.0
BES-anode	191,150	56	2.73	5.2
(BES→) FFR	198,254	170	4.31	11.3
(BES→) FFR-C	158,351	232	5.27	13.4
NBES-left	171,495	52	2.94	5.0
NBES-right	202,435	52	2.87	4.4
(NBES→) FFR	189,784	117	2.14	10.1
(NBES→) FFR-C	258,361	133	2.09	11.8

*OTUs* operational taxonomic units

**Fig. 3:**
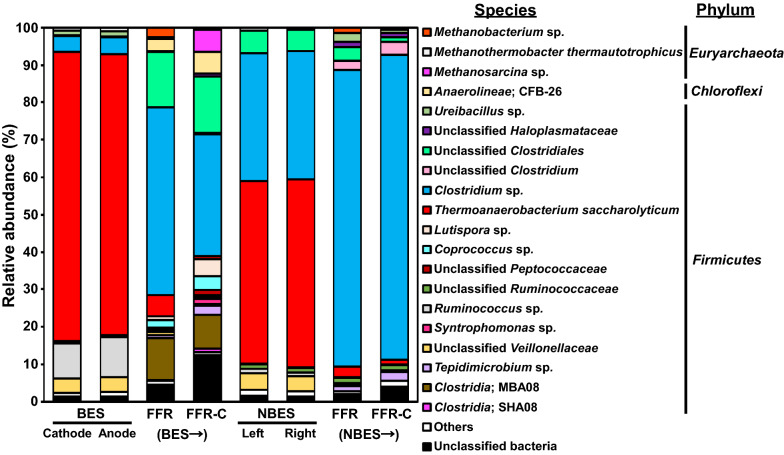
Species-level microbial diversity of microorganisms in the two-stage processes. The suspended cultures in the cathodic and anodic sides of the BES, or the left and right sides of the NBES, with the (BES→ and NBES→) FFR processes, or in the CFT-attached fractions in the (BES→ and NBES→) FFR-C processes were determined. Species with low similarity (< 97%) and low abundance (< 1.0%) were included in the “unclassified bacteria” and “others” categories, respectively

Microbial compositions were examined at the species level (Fig. 3). Most of the microorganisms were assigned to three phyla (*Firmicutes*, *Chloroflexi*, and *Euryarchaeota*) in the suspended fractions of two two-stage processes (BES → FFR and NBES → FFR) and in the CFT-attached fractions of FFRs [(BES→ and NBES→) FFR-C]. The dominant phylum was *Firmicutes* in the BES and NBES, consisting mainly of bacteria related to *Thermoanaerobacterium saccharolyticum*. Bacteria related to *Clostridium* sp. and *Ruminococcus* sp. constituted communities of predominant microorganisms in the BES, and bacteria related to Unclassified *Clostridiales* constituted communities of predominant microorganisms in the NBES. Almost no methanogens were detected in the BES and NBES, due to the low pH. In the suspended and CFT-attached fractions following the (BES→ and NBES→) FFR processes, bacteria related to *Clostridium* sp. constituted the major microbial community and were most abundant in (NBES→) FFR, corresponding to a low microbial diversity. With the (BES→) FFR process, bacteria related to unclassified species in the *Clostridiales* and MBA08 orders of *Clostridia* were the other predominant microorganisms. Interestingly, the relative abundances of methanogens related to *Methanosarcina* sp. increased after the (BES→) FFR-C process, compared to those found after the (NBES→) FFR-C process. The relative abundances of methanogens related to *Methanobacterium* sp. were higher in the suspended fraction of the (BES→) FFR process than those in the (NBES→) FFR process.

### Application of a current stabilizes the two-stage BES → FFR process

We aimed to investigate whether application of a current as a bioelectrochemical-preprocessing step would improve the CH_4_ yield of the two-stage NBES → FFR process. First, we operated each stage in the two-stage NBES → FFR process at an HRT of 2.5 and 4.2 days, respectively, after acclimatization (Fig. 1 and Table 1). Next, an electric current was applied during the BES stage, followed by the FFR stage (BES → FFR process), which were performed with HRTs of 2.5 and 4.2 days, respectively, after acclimatization. The outlet pH values were 5.6 ± 0.1, 6.3 ± 0.1, 5.2 ± 0.1, and 6.6 ± 0.1 with the NBES, (NBES→) FFR, BES, and (BES→) FFR processes, respectively.

**Fig. 4:**
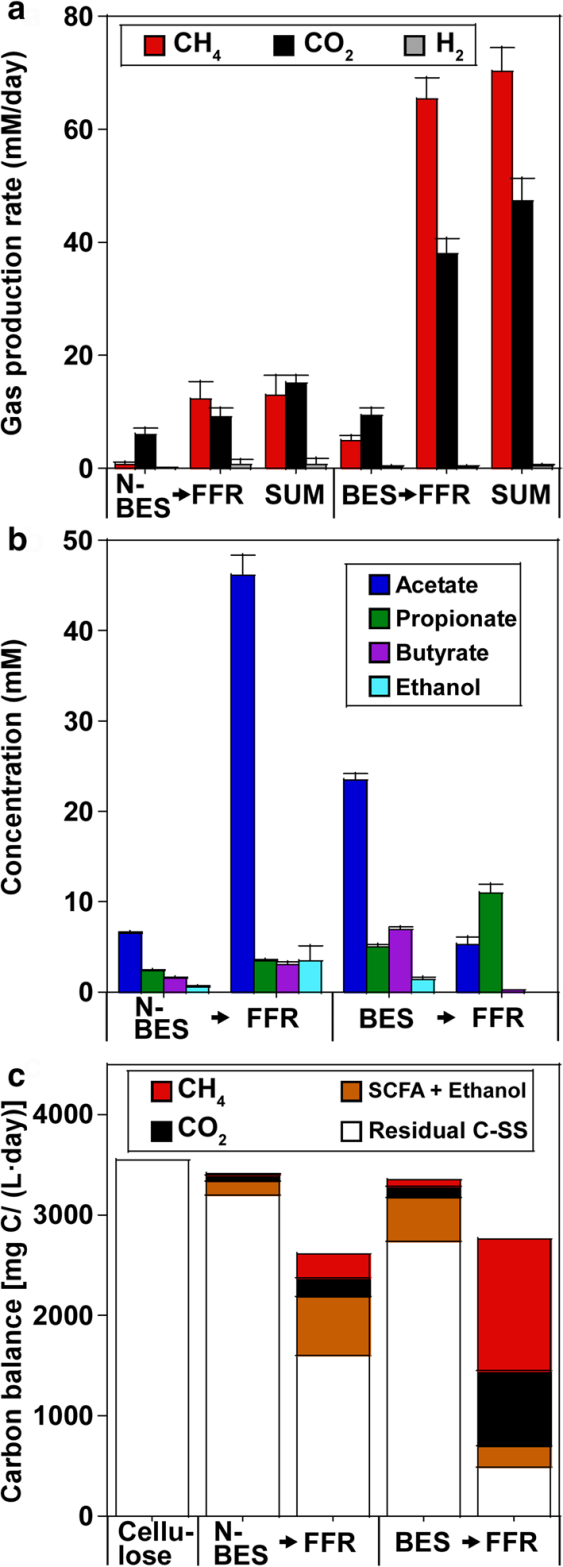
Performances of the two-stage NBES → FFR and BES → FFR processes using HRTs of 2.5 → 4.2 days. **a** Rates of gas production in terms of CH_4_, CO_2_, and H_2_. **b** Production of SCFAs and ethanol. **c** C balances of the two-stage processes. The cellulose loads of the BES and NBES were both 3555.6 mg C/(L day). The residual C in all reactors was calculated based on the SSs

Initially, SS removal in the NBES was 10.0 ± 0.1%, and as expected, SS removal was only 49.9 ± 4.2% in the following FFR (Fig. 4). Interestingly, due to applied current in the first stage, SS removal increased to 82.3 ± 3.0% in the (BES→) FFR process, whereas SS removal in the BES was 23.2 ± 0.9%. Accordingly, biogas production was low (total 22.1 ± 3.8 mM/day) in the (NBES→) FFR process; however, biogas production increased (total 103.3 ± 3.9 mM/day) in the (BES→) FFR process (Fig. 4a, c). High quantities of SCFAs and ethanol (total 56.1 ± 1.8 mM) accumulated during the (NBES→) FFR process; however, these values decreased (total 16.5 ± 1.9 mM) in the (BES→) FFR process (Fig. 4b, c). As a result, the two-stage NBES → FFR process only demonstrated a CH_4_ yield of 7.1 ± 1.9% with 47.9 ± 1.9 vol% CH_4_ in the product gas. By applying current during the first stage, the two-stage BES → FFR process showed an enhanced CH_4_ yield of 38.4 ± 2.3% with 60.8 ± 2.4 vol% CH_4_ in the product gas.

### CFTs are necessary for stable operation of the two-stage BEC → FFR process

**Fig. 5:**
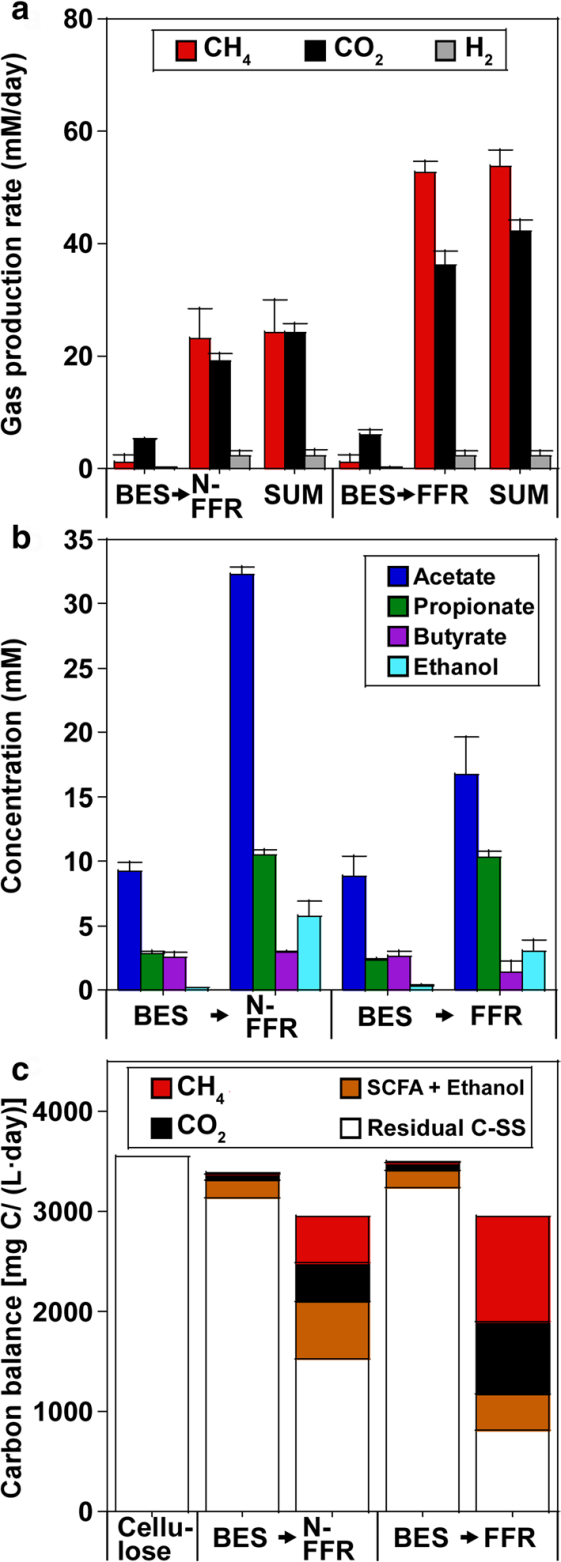
Performances of the two-stage BES → NFFR and BES → FFR processes using HRTs of 2.5 → 4.2 days. **a** Rates of gas production in terms of CH_4_, CO_2_, and H_2_. **b** Production of SCFAs and ethanol. **c** C balances of the two-stage processes. The cellulose loads of the BES and NBES were both 3555.6 mg C/(L day). The residual C in all reactors was calculated based on the SSs

We ascertained the necessity of a conductive material, CFT, in the second stage (FFR). First, we operated a two-stage process, namely BES and NFFR without CFTs (BES → NFFR) at HRTs of 2.5 and 4.2 days, respectively, after acclimatization (Fig. 1 and Table 1). Next, CFTs were installed in the second stage, and we operated BES and subsequent FFR (BES → FFR) at an HRT of 2.5 and 4.2 days, respectively, after acclimatization. The outlet pH values were 5.8 ± 0.4, 6.8 ± 0.0, 5.4 ± 0.4, and 6.6 ± 0.1 in the BES, (BES→) NFFR, another BES, and (BES→) FFR processes, respectively. At first, SS removal in the BES was 11.9 ± 0.6%, as observed in other BESs; however, SS removal was only 51.2 ± 1.6% following (BES→) NFFR (Fig. 5). Interestingly, after installation of CFTs into the NFFR, SS removal increased to 74.9 ± 5.7% in (BES→) FFR, whereas SS removal in the BES was 8.9 ± 1.5%. Accordingly, biogas production was low (total 44.4 ± 5.8 mM/day) in the (BES→) NFFR process; however, biogas production increased (total 91.0 ± 1.8 mM/day) in the (BES→) FFR process (Fig. 5a, c). High amounts of SCFAs and ethanol (total 51.5 ± 1.9 mM) accumulated in the (BES→) NFFR process; however, these values decreased (total 31.4 ± 5.1 mM) in the (BES→) FFR process (Fig. 5b, c). As a result, the two-stage process, BES → NFFR, showed a CH_4_ yield of 13.4 ± 3.1% with 48.8 ± 5.8 vol% CH_4_ in the product gas. By adding CFT in the second stage, the two-stage BES → FFR demonstrated an enhanced CH_4_ yield of 30.0 ± 1.4% with 56.0 ± 2.6 vol% CH_4_ in the product gas.

## Discussion

In this study, we developed a thermophilic two-stage process consisting of BES and anaerobic digester-fixing CFTs. Conversion of cellulose to CH_4_ by microorganisms was enhanced using this type of two-stage process, compared to the process without an external electrochemical system in the first stage or without CFT in the second stage. We compared the input energy in terms of the applied electric current with the energy of CH_4_ produced by microorganisms. CH_4_ can be produced electrochemically through carbon dioxide (CO_2_) reduction [19] via the following reaction:

$$ {\text{CO}}_{{2}} \, + \,{\text{8H}}^{ + } \, + \,{\text{8e}}^{-} \, \to \,{\text{CH}}_{{4}} \, + \,{\text{2H}}_{{2}} {\text{O}}, $$

where 8 mol of electrons, that is 771,882 C (eight times the Faraday constant), are needed to produce of 1 mol of CH_4_. In the BES, 8.64 C (= 100 × 10^–6^ × 86,400 s) of current per L was used for 1 day. If this was utilized entirely for CH_4_ production, a maximum of 0.27 mM/day of CH_4_ should be produced electrochemically under standard conditions. However, we obtained considerable amounts of CH_4_ (53.9–70.2 mM/day) with our two-stage process containing BES. Evidently, a small amount of electric current affected the activity of the microbial community in our two-stage process.

Bacteria related to *Clostridium* sp., *T. saccharolyticum*, *Ruminococcus* sp., and unclassified *Veillonellaceae* were predominant during the first stage (BES or NBES), after loading with cellulose (Fig. 3). Cellulose is highly recalcitrant to biodegradation, although cellulolytic microorganisms have been reported from the strains of *Clostridium* spp. and *Ruminococcus* spp. [20]. Additionally, uncultured *Clostridia* MBA08, mainly found with the (BES→) FFR process, was previously discovered in a thermophilic laboratory-scale digester used to process municipal solid waste [21]. Bacteria, such as *Clostridium* sp. and *Ruminococcus* sp., were present during both the first stage (BES or NBES) and the second stage (FFR) and potentially consumed cellulose. Members of the Veillonellaceae family were present in paddy soil amended with rice straw [22], and these microorganisms utilized cellulose degradation products. Cellulose biodegradation was limited in the first stage, probably due to the short HRT of 2.5 days. *T. saccharolyticum* can ferment a wide array of carbohydrates, including starch, glucose, xylose, and arabinose, but it cannot degrade cellulose [23], and *T. saccharolyticum* present in the former and latter stages would expect to utilize carbohydrates other than cellulose.

Notably, the relative abundances of the H_2_-utilizing methanogen, *Methanobacterium* sp. [24], increased in the suspended fraction and acetate-utilizing methanogen, *Methanosarcina* sp. [25], increased in the CFT-attached fraction of the (BES→) FFR process. It should be taken into consideration that thermophilic *Methanosarcina* sp. can perform hydrogenotrophic methanogenesis [25]. Another hydrogenotrophic methanogen, *Methanothermobacter thermautotrophicus* [26], was present at low levels in both (BES→ and NBES→) FFRs. Data from our previous study showed that syntrophy of cellulolytic bacteria with hydrogenotrophic methanogens accelerated cellulose decomposition [27]. Thus, an increase in the abundance of H_2_-scavenging methanogens contributed to the efficient conversion of cellulose to CH_4_ in the (BES→) FFR process. The mechanism of this phenomenon in our two-stage process is discussed below. Data reported by Zhao et al. [28] indicated that a microbial electrolysis cell containing a conductive material (C felt) enhanced direct interspecies electron transfer to increase CH_4_ production. Additionally, supplementation of conductive materials, such as C cloth, shifted interspecies H_2_ transfer to direct interspecies electron transfer [29]. In this study, interspecies H_2_ transfer is expected to dominate in the second stage, when the FFR contained the conductive material, CFT, following the first (BES) stage. Interspecies H_2_ transfer would be promoted in both the first stage, when BES operated in microbial electrolysis cell mode (but abiotic H_2_ production was prevented) and in the latter stage, with the (BES→) FFR process. That is, the applied electrical current in the initial BES stage would favor interspecies H_2_ transfer in the following FFR stage. Findings on the dominance of acetate-removing *Methanosarcina* sp. on CFTs agreed with those of our previous study showing that *Methanosarcina* sp. were enriched on CFT because of the enhanced retention of these methanogens [30].

The abundance of suspended microbes and the microbial diversity in the CFT-attached fraction were higher in the (BES→) FFR system than those in the (NBES→) FFR system. Bacteria related to *Anaerolineae* CFB-26, *Lutispora* sp., *Coprococcus* sp., and *Syntrophomonas* sp. were particularly abundant in the (BES→) FFR system, probably due to the application of current to the first reactor. *Anaerolineae* demonstrate versatile metabolic abilities in terms of carbohydrate fermentation, but do not present any cellulolytic ability [31]. Additionally, *Coprococcus* can ferment carbohydrate [32]. *Lutispora thermophila* was capable of utilizing some amino acids for growth in the presence of yeast extract, but carbohydrates were not utilized [33]. The above-mentioned microorganisms produce acids. Bacteria, such as *Syntrophomonas,* can oxidize butyrate in syntrophic cooperation with methanogenic archaea [34]. These results are consistent with those of a previous research showing the accelerating effect of increasing microbial diversity on CH_4_ production with laboratory anaerobic digesters [35]. Ecosystems with higher microbial diversity would have a higher probability of functional redundancy or functional stability [36]. Our data suggest that increased microbial concentration and diversity contributed to the efficient microbial conversion of cellulose to acids. Additionally, efficient removal of acids with the aid of methanogens provided an environment that increased biomass and biodiversity in the reactor.

## Conclusions

In this study, we operated a thermophilic BES in microbial electrolysis cell mode (with a low current and without abiotic H_2_ production) using low-cost C sheets as the cathode and anode in the first stage. In the second stage, we operated a thermophilic FFR containing the conductive material, CFT. This type of two-stage process, BES → FFR, accelerated the decomposition of cellulose by microorganisms and stimulated CH_4_ production by decreasing the accumulation of acids and ethanol, when compared to the two-stage process without an electrochemical system (NBES → FFR) or without CFT (BES → NFFR). Electrochemical CH_4_ produced due to the applied current was negligible, compared to the overall level of CH_4_ production. Thus, both the electrochemical system and CFT were necessary for stable operation of the BES → FFR. The abundances of suspended microbes and the associated microbial diversity were higher in the (BES→) FFR process than in the (NBES→) FFR process. Bacteria with cellulolytic and saccharolytic abilities were present in the BES, NBES, and following (BES→ and NBES→) FFRs processes. Moreover, H_2_-consuming methanogens (*Methanobacterium* sp. and *Methanosarcina* sp.) showed increased relative abundances in the suspended fraction and attached fraction of the (BES→) FFR process, respectively, compared to those in the (NBES→) FFR process. Thus, interspecies H_2_ transfer was promoted in the FFR to accelerate the conversion of cellulose to CH_4_ by bioelectrochemical preprocessing. These results suggest that the BES → FFR process represents an advantageous system for the anaerobic digestion of cellulose, with a short HRT with the BES, thereby implying that BES and FFR can be subjected to small- and large-scale operations, respectively.

## Methods

### Configurations of the BES → FFR and NBES → FFR processes

BES was used for the first stage of fermentation. As shown in Fig. 1, BES was designed to have an H-type two-glass reactor (working volume of each reactor: 250 mL; total working volume: 500 mL) and a three-electrode system. Both the working electrode (cathode) and counter electrode (anode) were composed of C sheets (graphite blocks with dimensions of 25 mm × 75 mm × 2 mm), as shown in Fig. 1a. An Ag/AgCl reference electrode (saturated KCl) was inserted at the cathodic working side. Two C sheets were fixed in each reactor using stainless steel wire and connected to a potentiostat (PS-08; Tohogiken, Japan). The current of the working electrode was electrochemically regulated to 2.7 μA/cm^2^. Each glass reactor had one gas outlet port connected to a gas sampling collection bag. In contrast, the C sheets were allowed to remain open in the NBES under open-circuit operation and served as a control reactor for the BES (Fig. 1b).

FFR was used for the second stage after the BES or NBES. Each FFR was designed to have a glass reactor (working volume: 250 mL) equipped with two CFT sheets (DONACARBO paper; diameter: 25.0 mm; height: 70.0 mm; thickness: 2.4 mm; Osaka Gas Chemical Co., Ltd., Osaka, Japan) as support material. This glass reactor was connected to a gas sampling collection bag, as described above.

### Start-up and operation

The BES or NBES were inoculated with 500 mL of sludge from the CH_4_ fermenter and allowed to degrade 1% glucose at 55 °C. Next, the following FFR or NFFR was inoculated with 250 mL of the above-mentioned sludge. After inoculation, a predetermined volume of the fermentation liquid in both the cathodic and anodic sides of the BES or both the left and right sides of the NBES was discharged through the sampling port [37], and the same volume of fresh artificial medium was added once a day. The artificial medium contained the following (in deionized water): 20 g/L cellulose powder (Code 07748-75, Nacalai Tesque, Kyoto, Japan), 0.1 g/L KH_2_PO_4_, 0.2 g/L K_2_HPO_4_, 1 g/L yeast extract, 2 g/L NaHCO_3_, 1 g/L NH_4_Cl, 0.1 g/L MgCl_2_⋅6H_2_O, 0.1 g/L CaCl_2_⋅2H_2_O, 0.6 g/L NaCl, 10 mL/L trace element solution (Deutsche Sammlung von Mikroorganismen und Zellkulturen [DSMZ] 141 medium), and 1 mL/L vitamin solution (DSMZ 141 medium). A predetermined volume of fermentation liquid in the subsequent FFR or NFFR stage was discharged, and the same volume of the above-mentioned effluent (mixed in the cathodic and anodic sides, or the left and right sides) of the BES or NBES was added once a day. The contents of both the cathodic working and the anodic counter sides in the BES, or the left and right sides in the NBES, were mixed thoroughly using a magnetic stirrer before added to the FFR. The HRT was determined as the time required to replace the entire working volumes in the reactor. The temperature of the culture was maintained at 55 °C. The experiments were performed in triplicate.

### Analysis

The volume of gas produced was measured with a water displacement method using a graduated cylinder. The CH_4_, CO_2_, and H_2_ contents of the gas were measured using a gas chromatograph equipped with a thermal conductivity detector (GC390B; GL Sciences, Tokyo, Japan) and a stainless steel column packed with active C (30/60 mesh; GL Science), as per methods described previously [37]. The concentrations of lactate, acetate, propionate, butyrate, and ethanol were measured using high-pressure liquid chromatography (Shimadzu, Kyoto, Japan) with an Aminex HPX-87H column (Bio-Rad Laboratories, Hercules, CA, USA) and a RID-10A refractive index detector (Shimadzu). The suspension was filtered through a glass fiber filter (0.45 μm) to determine the SS contents in the cultures; then, the residue on the fiber was dried at 105 °C for 120 min, and the dry weight was measured. The removal of SS was determined using the following formula by considering the weight difference before and after fermentation:

$$ {\text{SS}}_{{{\text{removal}}}} \, \left( \% \right)\, = \,\frac{{{\text{SS}}_{{{\text{inlet}}}} {\text{ (g) }}{-}{\text{ SS}}_{{{\text{outlet}}}} {\text{ (g)}}}}{{{\text{SS}}_{{{\text{inlet}}}} {\text{ (g)}}}}, $$

where SS_inlet_ is the loaded SS weight and SS_outlet_ is the residual SS weight after fermentation. Residual C in SSs was calculated by multiplying the SS weight by 72/162, which reflects the molecular weight of 6 C atoms divided by the molecular weight of cellulose, assuming that the amount of microorganisms are much lower than the residual cellulose.

The yield of CH_4_ was calculated on the basis of g of C, as follows:

$$ {\text{CH}}_{{4}} {\text{yield }}\left( \% \right)\, = \,\frac{{\text{g of C in CH4}}}{{\text{g of C in cellulose put into the reactor}}}. $$

### Microbial DNA extraction

Whole genomic DNA was prepared from the cultures and CFT-attached fractions, as per methods described previously [37]. A 5000-μL aliquot of each culture was centrifuged at 5000×*g*, and the pelleted material was suspended in 200 μL of Tris-ethylenediaminetetraacetic acid (EDTA) buffer (10 mM Tris–HCl, 1 mM EDTA, pH 8.0). Alternatively, the attached fraction was vortexed and recovered in 5000 μL of Tris–EDTA buffer, and then the pelleted material after centrifugation was suspended in 200 μL of Tris–EDTA buffer, as described above. Each of these suspended materials was transferred to a sterilized and DNA-free bead-beating tube containing 300 mg of glass beads (diameter: 0.1 mm). Five hundred microliters of Tris–EDTA-saturated phenol, two hundred fifty microliters of lysis buffer, and fifty microliters of 10% (w/v) sodium dodecyl sulfate were added to each tube. The mixtures were then shaken vigorously for 30 s at 5.0 m/s using the FastPrep-24 instrument (MP Biomedicals, USA). Next, each mixture was centrifuged at 22,000×*g* for 5 min. Each upper aqueous layer was transferred to a fresh tube containing 275 μL of isopropyl alcohol and a 1/10 volume of 3 M sodium acetate, and then chilled at − 20 °C for 10–15 min. The extracted DNA precipitate was pelleted by centrifugation at 22,000×*g* for 5 min, washed with 70% ethanol, and then dried under a vacuum. The DNA was subsequently dissolved in Tris-EDTA buffer.

### Microbial community analysis

The V3–V4 region of the prokaryotic 16S rRNA gene was amplified using genomic DNA as the template and the primers Pro341F (5′-CCTACGGGNBGCWSCAG-3′) and Pro805R (5′-GACTACNVGGGTATCTAATCC-3′) [38], where N, B, W, and V correspond to degenerate nucleotides A/C/G/T, G/T/C, A/T, and A/C/G, respectively, as per previously described methods [37]. PCR and amplicon pool preparation were performed according to the manufacturer’s instructions (Illumina, San Diego, CA, USA). PCR amplicons were purified using AMPure XP DNA purification beads (Beckman Coulter, Brea, CA, USA) and eluted in 25 μL of 10 mM Tris (pH 8.5). Purified amplicons were quantified using the Agilent Bioanalyzer 2100 with DNA 1000 chips (Agilent Technology, Santa Clara, CA, USA) and the Qubit 2.0 instrument (Thermo Fisher Inc., Waltham, MA, USA), and pooled at equimolar concentrations (5 nM). The 16S rRNA genes and an internal control (PhiX control v3; Illumina) were subjected to paired-end sequencing using the MiSeq instrument (Illumina) and the MiSeq Reagent Kit, v3 (600 cycles; Illumina). The PhiX sequences were removed, and paired-end reads with *Q* scores ≥ 20 were joined using the Automated CASAVA 1.8 paired-end demultiplexed fastq, which was performed with the FASTQ Generation program on the Illumina Basespace Sequence Hub (https://basespace.illumina.com/). Sequence quality control and feature table construction of the sequence data were performed and corrected using QIIME 2 version 2018.2 (https://qiime2.org) and the DADA2 pipeline [39]. The taxonomic compositions of OTUs were classified using the Naive Bayes classifier. This classifier was trained on the Greengenes 13_8 99% OTUs full-length sequence database (https://data.qiime2.org/2018.2/common/gg-13–8-99-nb-classifier.qza). The OTU data were used for α-diversity estimation of the Faith’s phylogenetic diversity [40] and Shannon’s indices [41, 42].

### Real-time PCR analysis

Real-time PCR was performed to quantify total bacteria, using the LightCycler 96 system (Roche, Basal, Switzerland) with a universal primer set (5′-ACTCCTACGGGAGGCAGCAGT-3′ and 5′-GTATTACCGCGGCTGCTGGCAC-3′) targeting eubacteria [43]. PCR amplification was performed as per methods described previously [44].

## Data Availability

The datasets supporting the conclusions of this study are available in the MG-RAST server (https://www.mg-rast.org) [45] in a database titled “Two-stage hydrogen/methane fermentation process by bioelectrochemical system” under accession numbers mgm4892919.3–mgm4892926.3.

## Abbreviations

BES: Bioelectrochemical system
C: Carbon
CFT: Carbon fiber textile
CH_4_: Methane
CO_2_: Carbon dioxide
DSMZ: Deutsche Sammlung von Mikroorganismen und Zellkulturen
EDTA: Ethylenediaminetetraacetic acid
FFR: Fixed film reactor
H_2_: Hydrogen
HRT: Hydraulic retention time
NBES: Non-bioelectrochemical system
NFFR: Non-fixed film reactor
OTU: Operational taxonomic unit
pH_in_: Inlet pH
RNA: Ribonucleic acid
PCR: Polymerase chain reaction
SCFA: Short-chain fatty acid
SS: Suspended solid
SS_inlet_: Loaded SS weight
SS_outlet_: Residual SS weight after fermentation
